# An assessment of the water use of hemp: a scoping review and bibliometric analysis

**DOI:** 10.1186/s42238-025-00370-z

**Published:** 2025-12-01

**Authors:** S. Gokool, S. Mantel, A. Clulow, R. Kunz, A. Palmer

**Affiliations:** 1 Centre for Water Resources Research, School of Agriculture and Science, University of KwaZulu-Natal, Pietermaritzburg, 3209 South Africa; 2 Institute for Water Research, Rhodes University, PO Box 94, Makhanda, 6140 South Africa; 3 Discipline of Agrometeorology, School of Agriculture and Science, University of KwaZulu-Natal, Pietermaritzburg, 3209 South Africa; 4 South African Environmental Observation Network (SAEON), Grasslands-Forests-Wetlands Node, Pietermaritzburg, 3201 South Africa

**Keywords:** Hemp, Water requirements, Evapotranspiration, Crop water productivity

## Abstract

Recently, there has been a renewed and rapidly growing interest globally in the cultivation of the non-psychoactive variety of *Cannabis* more commonly referred to as hemp. However, there remains a scarcity of available scientific information on the water use of hemp to optimally guide its large-scale production. To address this knowledge gap, eligible peer-reviewed publications acquired from the Scopus, Web of Science and Google Scholar abstract and citation databases, were analysed using Biblioshiny and VOSviewer to gain further insights on the water use of this crop. A key finding emanating from this scoping review and bibliometric analysis was that the water use of hemp ranged from approximately 220–700 mm throughout the growing season, with daily water use generally ranging between ~ 4.00 mm d^− 1^ to ~ 5.00 mm d^− 1^. Variations in water use were primarily due to factors such as climatic regime, meteorological conditions, irrigation and agricultural management practices. Furthermore, several studies demonstrated that hemp’s deep rooting system and its unique ability to regulate stomatal conductance and photosynthesis enable it to withstand water stress, increasing its resilience to drought. While these findings provide useful insights into the water use of hemp, there exists a need for further research across a broader range of agro-ecological zones, agricultural management practices and genotypes to gain a more comprehensive and objective understanding of the water use requirements associated with hemp cultivation. This may then facilitate legislation, regulatory frameworks and agricultural management practices to be developed and adapted accordingly to not only optimize hemp production but also to safeguard present and future water resources security.

## Introduction

The cultivation of *Cannabaceae* plant family species has been practiced throughout the world for centuries due to its multi-purpose nature. All parts of the *Cannabis* plant can be used and it is typically grown for fibre, seed and oil which are used for a variety of applications such as agriculture, clothing and textiles, paper and compressed wood products, alternative medicine, cosmetics, bioenergy, phytoremediation and carbon sequestration (Sawler et al., 2015; Irakli et al., 2019; Adesina et al., 2020; Zheng et al., 2021; Zydelis et al., 2022; Gill et al., 2023).

Considering its potential to cause psychogenic effects and be used as a drug (Zydelis et al., 2022), the legality of *Cannabis* cultivation does vary throughout the world, with its cultivation in some places being completely legal (or restricted to certain conditions), whereas in other regions it is prohibited (Kwiatowska et al., 2024). Within the *Cannabaceae* plant family, there are varieties (often referred to as marijuana) which contain a psychoactive compound known as tetrahydrocannabinol (THC) which can cause psychogenic effects. In contrast, other varieties contain low levels of THC (< 0.30%) which are non-psychoactive. The non-psychoactive variety of Cannabis (Hemp) is among the most widely and legally cultivated form of Cannabis globally, particularly for industrial purposes (Zydelis et al., 2022; Kwiatowska et al., 2024).

Despite the stigma and legalities associated with *Cannabi*s cultivation, there has been a renewed and rapidly growing interest globally in the cultivation of this plant, with legislation and regulatory frameworks around its cultivation continuously evolving. Presently, *Cannabis* is legally cultivated in many countries and is one of the most rapidly expanding agricultural sectors across the world. This has contributed to the legal cultivation of Cannabis growing to become a multi-billion-dollar industry (Malabadi et al., 2024). From a South African perspective, the potential socio-economic and environmental benefits have driven the debate around the legalisation of *Cannabi*s cultivation and have contributed to changing legal and regulatory requirements as well as an increasing drive to follow environmentally sustainable development pathways. While there is a general consensus that *Cannabi*s cultivation can economically benefit both commercial large-scale and informal small-scale growers, there is a need to better understand the environmental impacts of widespread *Cannabi*s cultivation within the country, particularly its impact on available water resources (Mantel et al., 2025).

South Africa is a largely semi-arid country with concerns regarding both present and future water resources security. It has often been stated that Cannabis is a water intensive plant. However, some studies (Drastig et al., 2020; Thevs and Nowotny, 2023; Gill et al., 2023) have shown that relative to many major commercially cultivated crops such as cotton, wheat, maize and sorghum, *Cannabi*s uses less water (Herppich et al., 2020). Additionally, Cannabis has also been shown to have higher crop water productivity (amount of biomass or yield generated per unit of water) than some of the aforementioned commercial crops, as well as being fairly resilient to the impacts of water stress (Ashworth and Vizuete, 2017; Zheng et al., 2021).

Despite these diverging views, much of the available information pertaining to *Cannabi*s’s water use is anecdotal, as there remains limited research and scientific evidence largely owing to its longstanding illicit status (Gill et al., 2023). In order to optimize the cultivation of hemp, particularly in water-limited environments, there is a need to better understand the physiological and morphological responses of hemp under differing climatic regimes and growing conditions as this has a significant influence on growth, development and yield of the crop (Drastig et al., 2020; Danzinger and Bersntein; 2021; Zheng et al., 2021).

Considering the scarcity of available scientific information on the water use of *Cannabi*s, the focus of this study was to conduct a systematic scoping review, to compile an up-to-date evidence base that consolidates all available scientific information, to better understand the water use of *Cannabi*s to potentially facilitate improved and informed agricultural and water resources management decisions to be made with regards to its cultivation.

## Materials and methods

The Preferred Reporting Items for Systematic Reviews and Meta Analysis (PRISMA) approach (Tricco et al., 2018) was used to guide the systematic review process which entails (i) conducting a rigorous search of the available literature, (ii) evaluating the literature to determine eligibility and reduce biases and (iii) an in-depth analysis of the literature. The Scopus, Web of Science and Google Scholar abstract and citation databases were used to identify literature that would eventually form the literature database used in the bibliometric analysis.

Google Scholar was used only for the identification of articles not included in the other abstract and citation databases. The structured query string used to identify literature within these abstract and citation databases was developed by first performing an initial search within Google Scholar to identify potential keywords and variants that could be used, as well as leveraging the authors’ personal experiences in the subject matter.

**Fig. 1:**
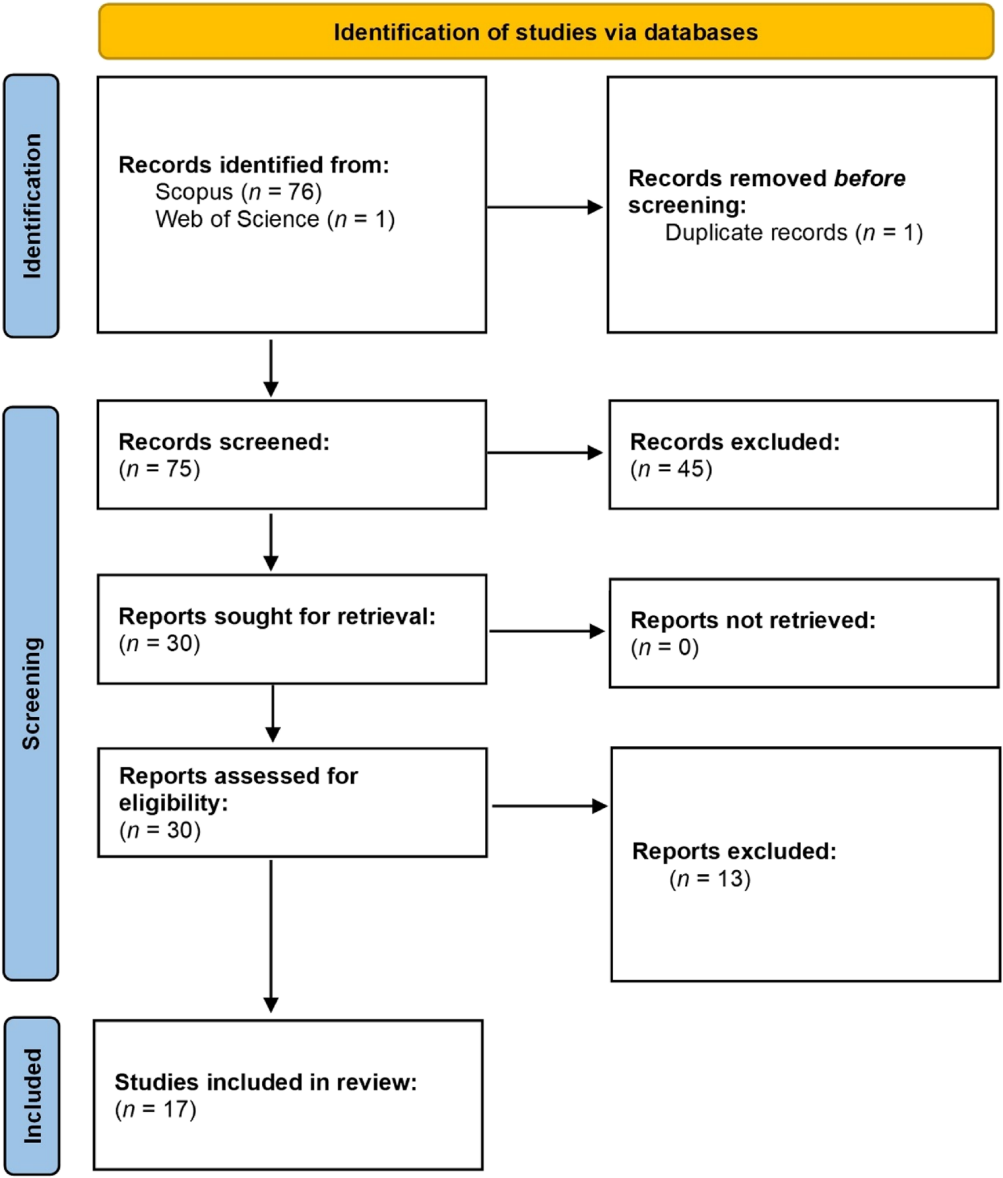
A flow diagram of the article selection process used to create the final literature database used in the bibliometric and attribute analysis

The following keywords and variants were used in a structured query string (“hemp” AND (“water use” OR “water requirements” OR “evapotranspiration” OR “transpiration” OR “water demand”)) to source the available literature on the 08th of May 2025. There were no restrictions applied to the search period, however, only articles written in English for which the full-text was available were sourced for further evaluation. A flow diagram of the article selection process to create the final literature database is shown in Fig. 1. In total, there were 76 (Scopus *n* = 75; Web of Science *n* = 1) articles available for further evaluation. The retrieved literature was then imported into the R statistical software (version 4.5.0) and 1 duplicate was removed using the bibilometrix package (Aria and Cuccurullo, 2017) prior to further screening. Three reviewers screened the title, abstract and keywords of the remaining 75 articles to establish the eligibility for inclusion in the review based on the following criteria:

- Only studies focusing on the measurement, estimation or reporting of hemp’s water use were considered, since this has generally been the most widely and legally cultivated form of *Cannabi*s globally and possesses fewer concerns around its potential use as a drug due to its non-psychoactive nature.
- The study either measured, attempted to estimate or reported on hemp water use.
- Studies that only reported on the amount of irrigation applied and not crop water use were not considered.

Following the screening, 45 articles were excluded as they did not meet the eligibility criteria. The remaining 30 articles were read in full to ensure they conformed to the eligibility criteria and a further 13 articles were removed. The bibliometric information of the remaining 17 articles was exported to the Biblioshiny and VOSviewer (version 1.6.20) software applications for further analysis.

## Results

**Fig. 2:**
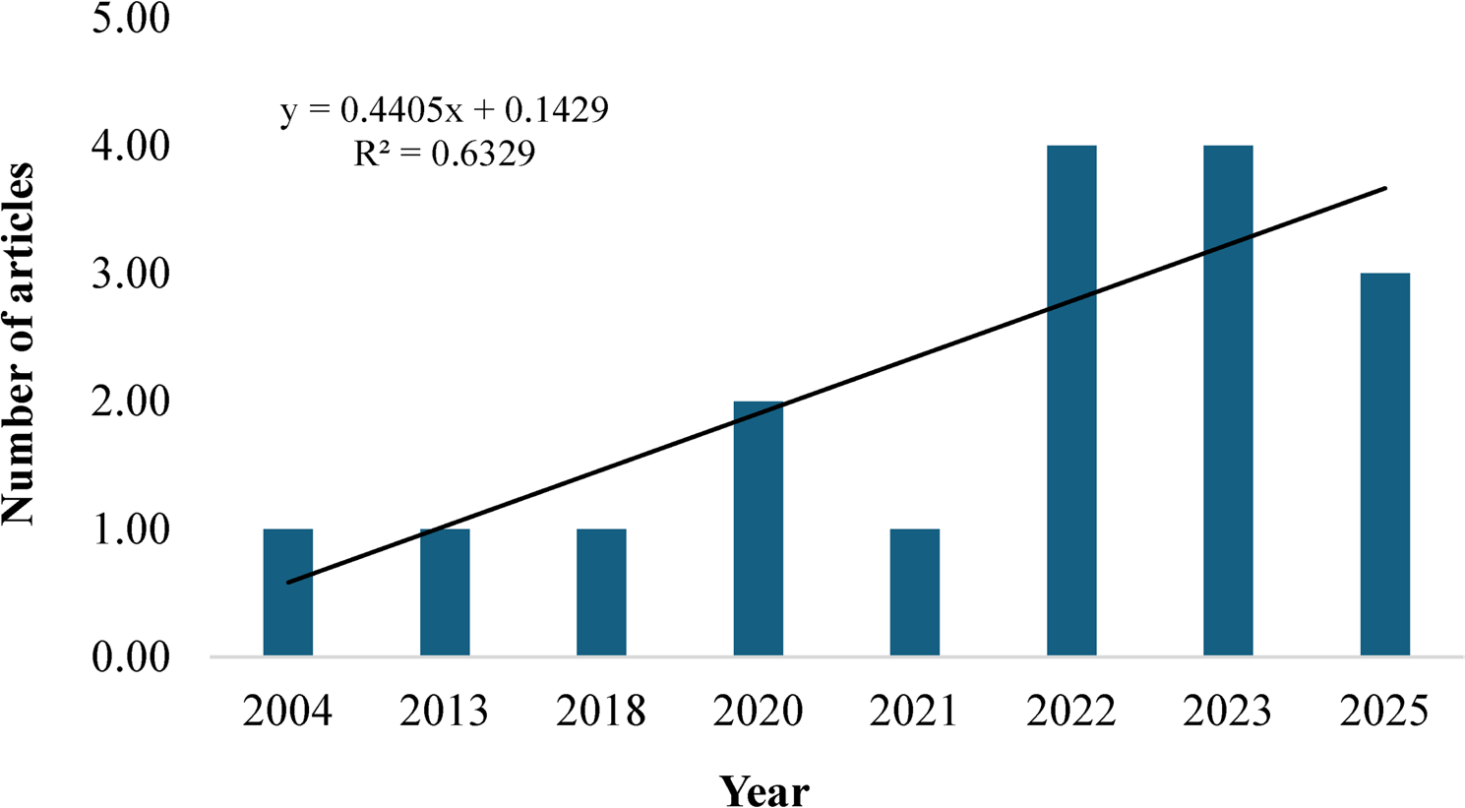
Historical evolution of the number of studies investigatinghemp’s wateruse

An overview of the key statistical information pertaining to the 17 studies comprising the final literature database is presented in Table 1, whilst Table 2 provides a summary of the key attributes across the various reviewed studies. From this literature database, research regarding the quantification of hemp water use first began in 2004 with the number of studies slowly but steadily increasing over time (Fig. 2). This reflects the growing interest in better understanding hemp’s water use, particularly within the past 5 years with greater than 80% of the studies being published during this period.

**Table 1:** A summary of the key bibliometric information contained within the final literature database

Description	Result	Description	Result
Timespan	2004–2025	References	871
Number of journals	15	Keywords plus (ID)	127
Number of publications	17	Author’s keywords (DE)	74
Annual growth rate	5.37	Authors	69
Average citations per document	18.24	Co-authors per document	5.12

**Table 2:**
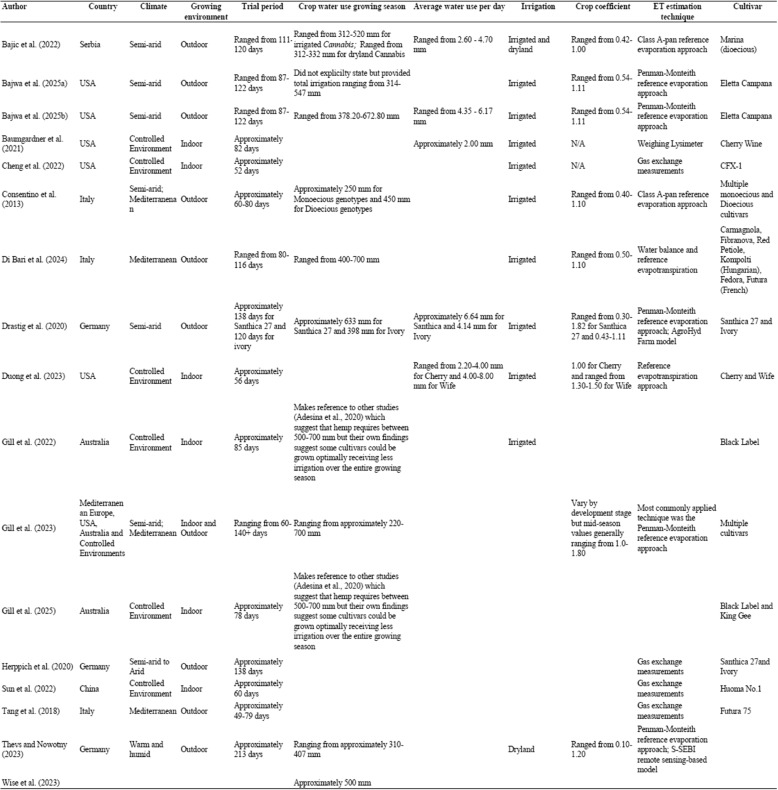
Summary of key attributes across the various reviewed studies

**Fig. 3:**
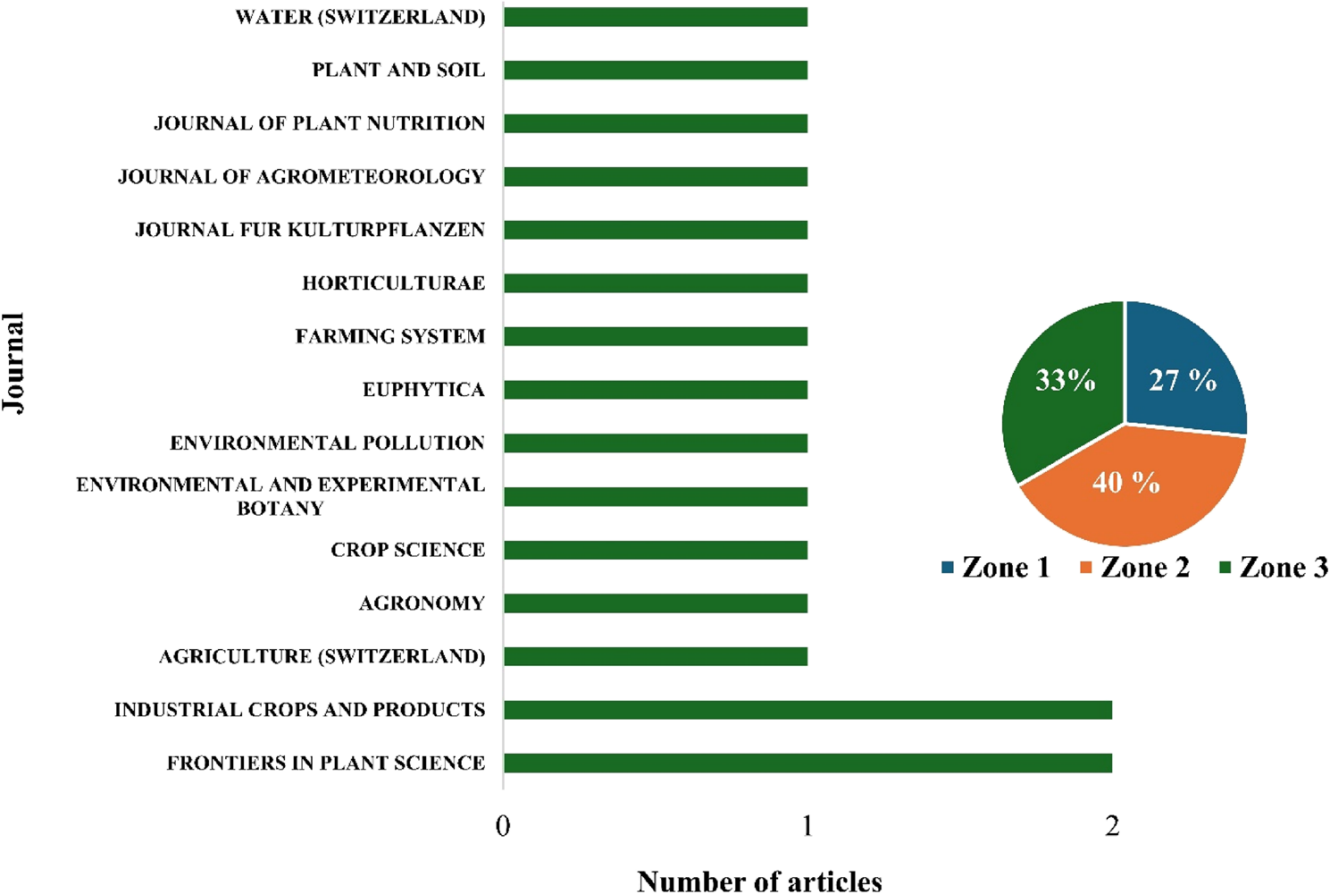
Number of articles per journal and classification according to Bradford’slaw

The 17 studies were published across 15 peer-reviewed academic journals. *Industrial Crops and Products* and *Frontiers in Plant Science* are the only journals to have published more than one study (Fig. 3) and are the most influential journals according to their number of citations (*n* = 107 and 51, respectively) and h-index (2).

They are also among the four journals to be classified within the Zone 1 category according to Bradford’s law (Abafe et al., 2022), which signifies the most prominent journals within a specific research focus area as they receive the greatest scholarly attention (highest frequency of views and citations), whereas journals classified in Zones 2 and 3 receive lesser attention due to the lower frequency of published articles within a specific research focus area.

A total of 69 authors contributed to the 17 studies of which 14 have authored multiple studies (Table 3). The study by Consentino et al. (2013) received the highest number of citations to date (*n* = 80), which evaluated the adaptive capacity and productivity of various monoecious and dioecious hemp genotypes under mediterranean climatic conditions in southern Italy. Their results demonstrated that both monoecious and dioecious hemp genotypes can achieve high levels of crop water productivity with approximately 250–450 mm of water required during the growing season for optimal growth.

**Table 3:** Author level citation metrics for authors with more than one publication pertaining to the water use of hemp

	Author	h-index	g-index	m-index	Total citations	Number of publications	Publication start year
BURTON RA		2	3	0.50	43	3	2022
CAVAGNARO TR		2	3	0.50	43	3	2022
GILL AR		2	3	0.50	43	3	2022
LOVEYS BR		2	3	0.50	43	3	2022
DRASTIG K		2	2	0.33	36	2	2020
FLEMMING I		2	2	0.33	36	2	2020
GUSOVIUS H-J		2	2	0.33	36	2	2020
HERPPICH WB		2	2	0.33	36	2	2020
PEJIĆ B		2	2	0.50	10	2	2022
BAJWA P		1	1	1.00	1	2	2025
KAFLE A		1	1	1.00	1	2	2025
SAINI R		1	1	1.00	1	2	2025
SINGH S		1	1	1.00	1	2	2025
TROSTLE C		1	1	1.00	1	2	2025

Sun et al. (2022) was the highest-ranking study with regards to average citations per year as well as the second most cited study in the final literature database. Sun et al. (2022) aimed to evaluate whether the symbiotic relationship between hemp and arbuscular mycorrhizal fungi could be leveraged to improve hemp’s ability to withstand cadmium stress and improve crop growth dynamics within contaminated soils. The findings of the study demonstrated that the inoculation of hemp with *Rhizophagus irregularis* mycorrhizal fungi enhanced hemp’s adaptability to cadmium stress through the improved regulation of photosynthetic gas exchange and chlorophyll fluorescence parameters.

This can serve as a promising phytoremediation approach to improve the productivity of cadmium stressed agricultural lands, particularly as hemp fibre production does not enter the food chain to potentially contribute to negative health effects.

The study by Bajwa et al. (2025a) had the highest normalized total citation score, which evaluated how the growth dynamics of industrial hemp (in west Texas, USA) was impacted by planting dates and densities. During their investigations, the seed planting times and concentrations were varied across three consecutive months (April-June) and the impact of these practices on crop water use, root development and distribution, as well as yield were monitored. Their experimental results revealed that planting earlier with higher planting densities or seeding densities can potentially improve productivity in water-stressed environments.

Among the reviewed studies, Gill et al. (2022) is the only one to rank within the top five across all citation performance metrics. The aim of this study was to understand and quantify the physiological, biochemical and morphological responses of hemp to water stress. Hemp plants subjected to well-watered, moderately-stressed and highly-stressed conditions within a greenhouse environment were monitored over an 85-day period. The results demonstrated that while there was a reduction in total yield due to water stress, the plants were able to survive and maintain seed production by improving their crop water productivity through the regulation of stomatal conductivity and transpiration. These findings highlight hemp’s ability to withstand water stress and its potential to grow in water-limited environments.

**Fig. 4:**
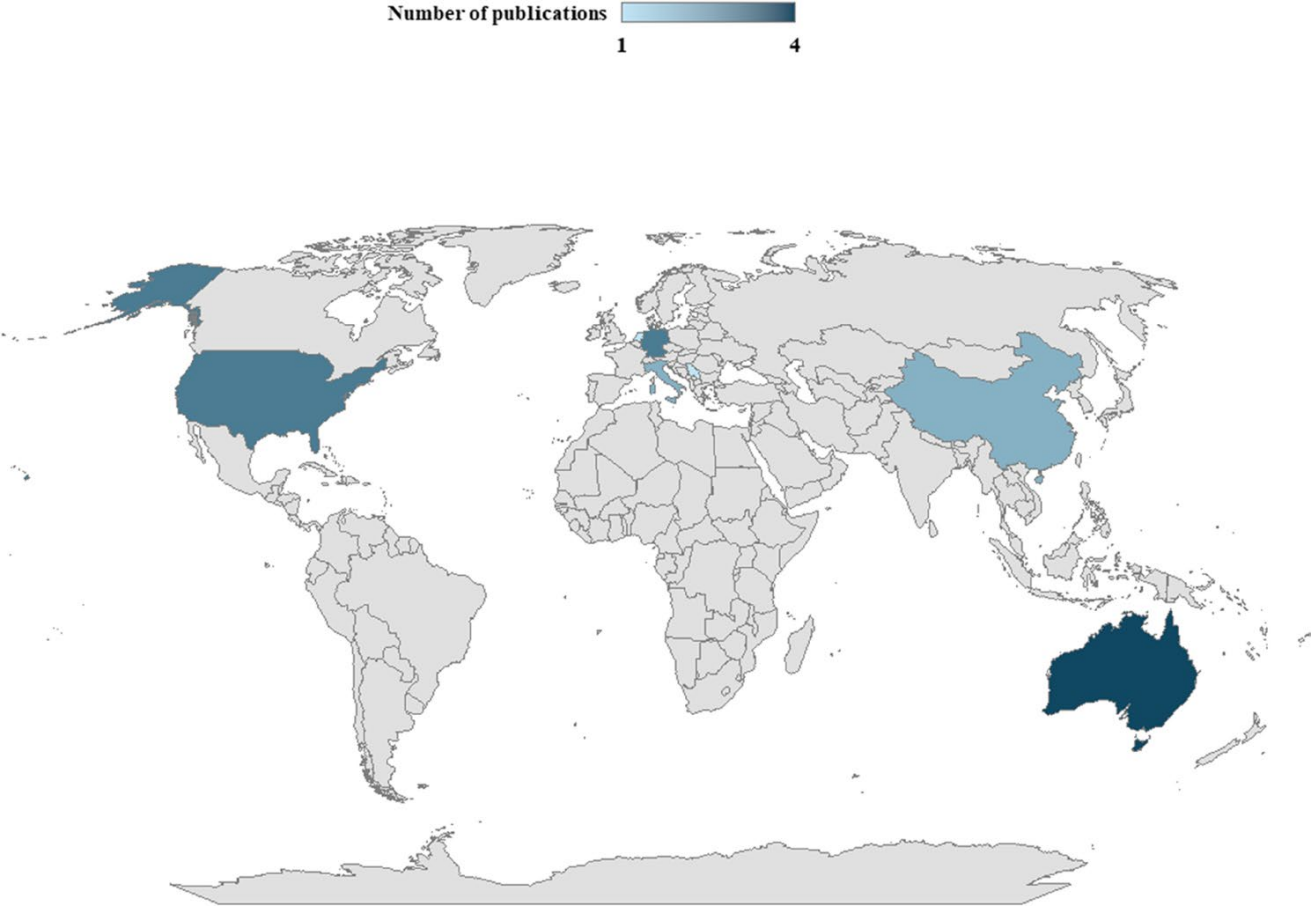
Geographic distribution of studies investigating the water use of hemp

The geographic distribution of all 17 studies (Fig. 4) shows that relatively few countries have conducted or participated in research pertaining to understanding and quantifying hemp’s water use, with approximately 70% of these studies occurring in developed countries.

**Fig. 5:**
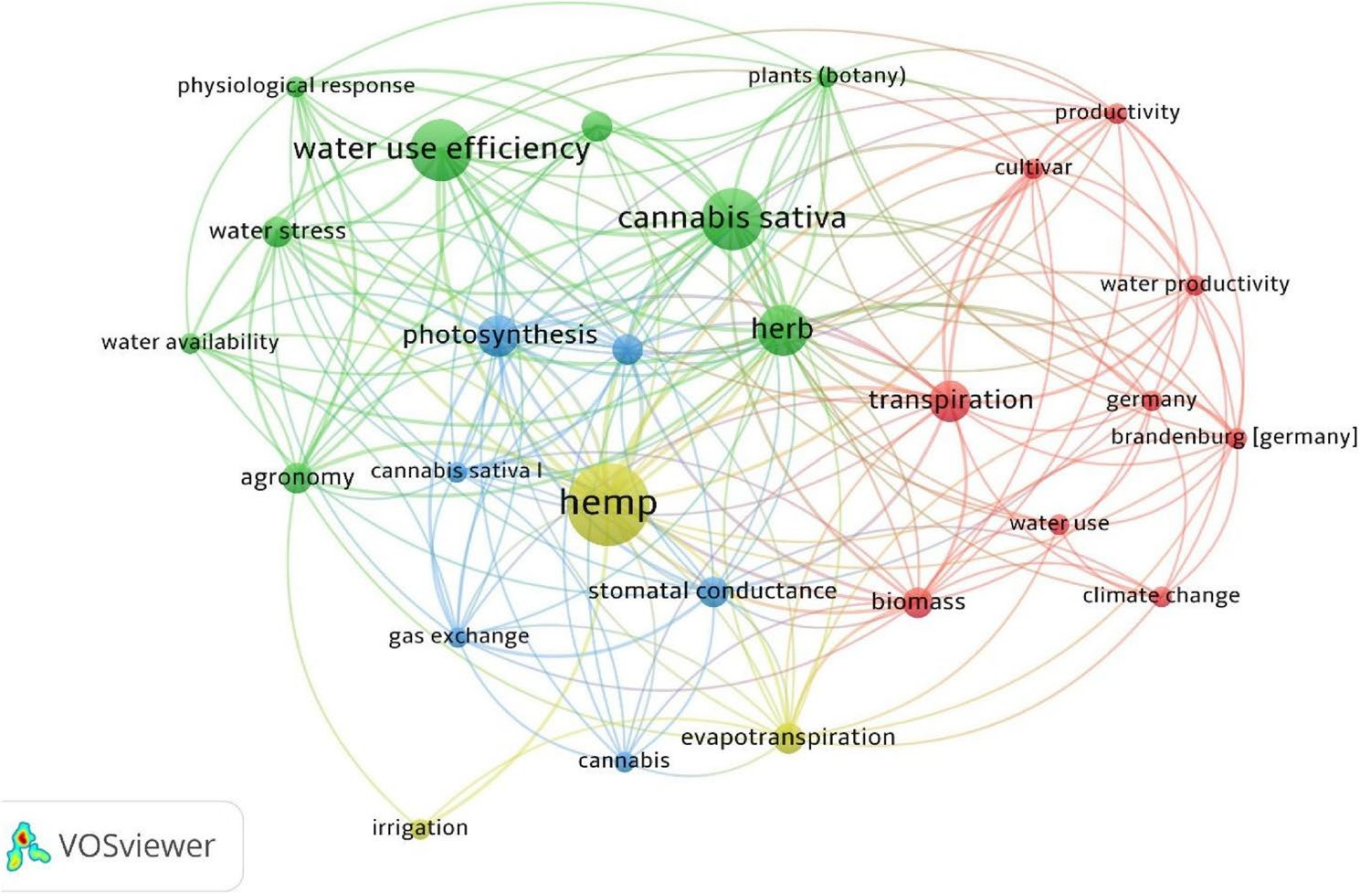
Co-occurrence network of all keywords

The most frequently used author keywords and keywords plus, as well as the co-occurrence of keywords that appear at least 2 times are shown in Table 4; Fig. 5, respectively. These results highlight that a strong focus of the studies was to understand and quantify the crop water productivity of hemp, particularly under water stressed conditions.

**Table 4:** Top 10 most frequent author keywords and keywords plus

Author Key words	Frequency	Author Key words +	Frequency
water use efficiency	6	water use efficiency	6
hemp	4	herb	5
cannabis sativa	3	cadmium	4
water stress	3	cannabis sativa	4
agronomy	2	hemp	4
evapotranspiration	2	photosynthesis	4
industrial hemp	2	stomatal conductance	4
irrigation	2	water stress	4
photosynthesis	2	biomass	3
agriculture	1	chlorophyll	3

## Discussion

In this study, we have attempted to evaluate all published literature pertaining to the water use of hemp to gain a better understanding of its water use requirements. Thereafter, all water use measurements and estimates were consolidated, unified and compared either for the entire growing season (mm per growing season) or in terms of daily water use (mm d^− 1^).

Seasonal water use across the various studies comprising the final literature database ranged from ~ 220 mm to ~ 700 mm per growing season (DiBari et al., 2004; Consentino et al., 2013; Drastig et al., 2020; Bajic et al., 2022; Gill et al., 2022; Gill et al., 2023; Thevs and Aliev et al., 2022; Thevs and Nowotny, 2023; Wise et al., 2023; Bajwa et al., 2025a, b; Gill et al., 2025). The seasonal water use requirements vary regionally due to several factors such as, the region’s biophysical characteristics (climate and soil), management practices (e.g., irrigation practices, mycorrhizal inoculation, planting dates and seeding density) as well as genotype (Zheng et al., 2021; Gill et al., 2022; Sun et al., 2022; Bajwa et al., 2025a, b). For example, Gill et al. (2022) reported that hemp generally requires between 500 and 700 mm of water throughout the growing season to achieve optimal yields, of which ~ 50% is required during the vegetative growing phase. Whereas, Consentino et al. (2013) indicated that hemp generally requires 250–450 mm of water throughout the growing season depending on genotype (monecious or dioecious).

From an average daily water use perspective, the water demand of *Cannabis* has often been reported or quoted as 22.70 L per day or approximately 6 gallons per day per plant (Zheng et al., 2021; Mtewa et al., 2024). This translates to approximately 9.77 mm d^− 1^ per plant, according to the water demand calculation reported in Zheng et al. (2021). This value appears to be an order of magnitude higher than the results from several studies and our own field experiments using eddy covariance and large aperture scintillometry (Mantel et al., 2025). The daily water use estimates across all 17 studies ranged from ~ 2.00 mm d^− 1^ to ~ 8.00 mm d^− 1^ (Baumgardner et al., 2021); Drastig et al., 2020; Bajic et al., 2022; Thevs and Aliev et al., 2022; Duong et al., 2023); Collado et al., 2024); Bajwa et al., 2025b) with values ranging between ~ 4.00 mm d^− 1^ to ~ 5.00 mm d^− 1^ being reported most frequently. These values are noticeably lower than the average daily water use of hemp that is often quoted (Zheng et al., 2021) and used as a basis for determining the water requirements of *Cannabis* throughout the growing season. Such discrepancies reaffirm the pressing need for more research into *Cannabis* water use so that objective and well-informed water resources and agricultural management decisions can be made with regards to its large-scale and widespread cultivation.

In order to better understand and more accurately quantify water use, it is important to adopt and utilize approaches that are able to adequately capture the spatio-temporal dynamics of *Cannabis* water use.

Several approaches were adopted in the reviewed studies which included in-situ methods such as portable gas exchange systems (primarily infrared gas analyzer measurements) and the reference evapotranspiration approach, as well as remote sensing-based methods. The reference evapotranspiration approach is one of the most common methods of quantifying crop water use as it requires relatively minimal inputs that are fairly easy to acquire. The technique utilizes meteorological data to estimate the evapotranspiration (ET) of a hypothetical well-watered reference surface (*ET*_*o*_) and then relates this water use to a crop of interest via a crop-coefficient (*K*_*c*_*)* which accounts for the crop’s unique biophysical characteristics (Allen et al., 1998; Thevs and Nowotny, 2023). Overall, *K*_*c*_ values utilized to estimate *Cannabis* water use ranged from 0.10 to 1.80, with values typically ranging from 0.40 to 0.50 for early-stages, 1.00–1.10 for mid-stages and 0.43–1.00 for late stages, respectively (Bajic et al., 2022; Thevs and Nowotny, 2023; Bajwa et al., 2025a, b). However, it should be noted that *K*_*c*_ values exceeding 1.40 are uncommon and only usually occur during advective conditions (Allen et al., 2011).

While portable gas exchange systems do provide accurate and reliable measurements of transpiration, these are generally for a few plants and scaling up these estimates to understand hemp’s water use across large geographic scales may prove to be challenging. Micro-meteorological approaches such as eddy covariance and scintillometry are to some extent better suited to overcome the aforementioned limitation but are also constrained by their spatial representativity, cost and labour requirements (Yacoob et al., 2024). The use of remote sensing-based methods (energy balance-based or vegetation index-based) as an alternate approach to acquire spatially and temporally representative water use estimates has shown a great deal of promise and has become more widely adopted in precision agriculture operations, particularly the use of spaceborne sensors, unmanned aerial vehicles and geospatial cloud computing (Yacoob et al., 2024) and are therefore potentially viable options to monitor and understand hemp’s water use across space and time. However, it should be noted that these techniques are generally limited to monitoring plants grown in outdoor settings.

In addition to reporting on hemp’s water use, a primary focus of the majority of the reviewed studies was on the crop water productivity (water use efficiency) of this crop, particularly during conditions of water stress (Table 4; Fig. 5). In these studies, hemp plants were subjected to some level of water stress through deficit irrigation practices and the water use, yield and biophysical responses to this stress were monitored.

The results of these investigations demonstrated that while total yield decreased, hemp was able to maintain a good reproductive capacity and produce reasonable yields, whilst also maintaining relatively good crop water productivity and being able to quickly recover from water stress (DiBari et al., 2004; Herppich et al., 2020; Gill et al., 2022; 2023; 2025).

Overall, the key findings highlighted in these studies demonstrated that hemp’s deep rooting system and its unique ability to regulate stomatal conductance and photosynthesis enable it to withstand water stress thereby increasing its resilience to drought (Thevs and Nowotny, 2023; Bajwa et al., 2025a; Gill et al., 2025). In addition to its general water use efficiency, hemp was also identified as a potentially feasible, lower water using alternate source of fibre for the textile industry. Wise et al. (2023) acquired data across 28 published studies to compare the water use of hemp against cotton which has traditionally been the predominant crop cultivated for fibre production for textiles. The results of their investigation indicated that hemp’s water use requirements and water footprint is significantly lower than cotton by approximately 38 and 60%, respectively. Similarly, Drastig et al. (2020) reported that cotton required between 763 and 915 mm of irrigation across the growing season which is higher than the water use of hemp reported across all the reviewed studies (~ 220 mm to ~ 700 mm). Since cotton is a relatively high-water use crop, the cultivation of hemp to supplement or replace cotton offers a potentially water efficient and more environmentally sustainable alternative to meet textile fibre production demands (Wise et al., 2023).

Considering that the cultivation of hemp has untapped economic potential for many large-scale and small-scale growers throughout the world, interest in the widespread cultivation of this crop will continue to grow. However, in order for this economic potential to be realized there is a need to improve and enhance the existing knowledge base regarding the optimal growth conditions for hemp production. This in turn will aid in guiding agricultural practitioners, large-scale and small-scale growers to better understand optimal growth requirements and evaluate whether the necessary resources are available to them to profitably cultivate this crop in an environmentally sustainable manner. Although this review has aided in consolidating the available scientific literature on the water use of hemp, the water use requirements of this crop as well as the physiological and morphological responses under varying water regimes is not yet well-understood.

Furthermore, several studies have shown that soil-related cultivation conditions such as nutrient supply and availability are crucial to the growth and development of the plant throughout its various phenological growth phases which in turn impacts its water use, crop water productivity and yield quality (Shiponi and Bernstein, 2021; Morad and Bernstein, 2023; Song et al., 2023; Kpai et al., 2024; Saloner et al., 2024). Therefore, future research which is aimed at better understanding both the optimal water and soil conditions will be key to optimizing and achieving high-quality yields across a range of environments and growing conditions.

## Conclusion

The multi-faceted nature of *Cannabis* and, in particular, hemp varieties has seen a renewed and growing interest in this crop due to the potential socio-economic and environmental benefits that can be derived from its cultivation. However, there is presently limited scientific research and information available regarding the water use requirements of this crop. *Cannabis* has often been described as a “water thirsty” plant.

Given the evolving nature of its legal status and regulatory requirements, the large-scale cultivation of hemp throughout the world is likely to increase. Therefore, it is imperative to better understand and quantify the water use of this crop to not only prevent the large-scale cultivation of this crop from becoming a threat to water resources security but to also optimize and improve agricultural productivity.

The findings presented in this review have shed some light on the water use of hemp, and thus can serve as a basis for guiding and informing future agricultural water management decisions with regards to the cultivation of this crop. With that being said, the findings herein are based upon a relatively limited body of scientific evidence with a specific focus on hemp. Therefore, it is evident that further research across a broader range of climatic and soil regimes, agricultural practices and genotypes is required to gain a more comprehensive and objective understanding of the water use associated with cultivating this crop. This will then enable legislation, regulatory frameworks and agricultural management practices to be developed and adapted accordingly to not only optimize *Cannabis* production but to also safeguard present and future water resources security.

## Data Availability

Data sharing is not applicable to this article as no datasets were generated or analysed during the current study.
